# Effects of Greater Central Arterial Stiffness on Cardiovagal Baroreflex Sensitivity in Resistance-Trained Men

**DOI:** 10.1186/s40798-021-00367-x

**Published:** 2021-10-26

**Authors:** Nobuhiro Nakamura, Isao Muraoka

**Affiliations:** 1 Faculty of Sport Sciences, Waseda University, Tokorozawa, Saitama Japan; 2 Faculty of Commerce, Yokohama College of Commerce, Yokohama, Kanagawa Japan; 3 Waseda Institute for Sport Sciences, Waseda University, Tokorozawa, Saitama Japan

**Keywords:** Cardiovagal baroreflex sensitivity, Central arterial stiffness, Central arterial compliance, Resistance training

## Abstract

**Background:**

Compared with age-matched untrained men, resistance-trained men who have undergone long duration training (> 2 years) at a high frequency (> 5 days/week) may be lower cardiovagal baroreflex sensitivity (BRS) because of central arterial stiffening. Therefore, the purpose of this study was to examine the effect of greater central arterial stiffness in resistance-trained men on cardiovagal BRS in a cross-sectional study to compare resistance-trained men with age-matched untrained men.

**Methods:**

This cross-sectional study included resistance-trained men (*n* = 20; age: 22 ± 3; body mass index: 26.7 ± 2.2) and age-matched untrained men (control group: *n* = 20; age: 25 ± 2; body mass index: 23.7 ± 2.4). The β-stiffness index and arterial compliance were assessed at the right carotid artery using a combination of a brightness mode ultrasonography system for the carotid artery diameter and applanation tonometry for the carotid blood pressure. And, the cardiovagal BRS was estimated by the slope of the R–R interval and systolic blood pressure during Phase II and IV of Valsalva maneuver (VM). The participants maintained an expiratory mouth pressure of 40 mmHg for 15 s in the supine position.

**Results:**

The β-Stiffness index was significantly higher in the resistance-trained group than in the control group (5.9 ± 1.4 vs. 4.4 ± 1.0 a.u., *P* < 0.01). In contrast, the resistance-trained group had significantly lower arterial compliance (0.15 ± 0.05 vs. 0.20 ± 0.04 mm^2^/mmHg, *P* < 0.01) and cardiovagal BRS during Phase IV of VM (9.0 ± 2.5 vs. 12.9 ± 5.4 ms/mmHg, *P* < 0.01) than the control group and. Moreover, cardiovagal BRS during Phase IV of VM was inversely and positively correlated with the β-stiffness index (*r* = − 0.59, *P* < 0.01) and arterial compliance (*r* = 0.64, *P* < 0.01), respectively.

**Conclusion:**

Resistance-trained group had greater central arterial stiffness and lower cardiovagal BRS Phase IV compared with control group. Moreover, the central arterial stiffening was related to cardiovagal BRS Phase IV. These results suggest that greater central arterial stiffness in resistance-trained men may be associated with lower cardiovagal BRS.

*Trial Registration* University hospital Medical Information Network (UMIN) in Japan, UMIN000038116. Registered on September 27, 2019.

## Key Points

- Cardiovagal baroreflex sensitivity (BRS) greatly contributes to the beat-to-beat control of arterial blood pressure through the autonomic nervous system and is positively associated with central arterial stiffness.
- Greater central arterial stiffness in resistance-trained men may be associated with lower cardiovagal BRS.
- Although the prescription of appropriate RT is highly recommended, this study raises caution regarding central arterial stiffening with RT.

## Background

The arterial baroreflex greatly contributes to beat-to-beat control of arterial blood pressure (ABP) through the autonomic nervous system. The cardiovagal baroreflex sensitivity (BRS) is blunted by aging [1–4]. Blunted cardiovagal BRS is a risk factor for life-threatening arrhythmias and is a predictor of sudden cardiac death [5, 6]. The cardiovagal BRS is correlated with age-related changes in central arterial mechanical properties such as stiffness and compliance where the baroreceptors are located (the carotid artery and the aortic arch) [2, 3, 7], because the arterial baroreceptors primarily sense the deformation of the arterial wall rather than intraarterial pressure changes [8]. Knutsen et al. [9] have suggested that the arterial diameter experiences less distension in a stiffer artery. Thus, to maintain appropriate cardiovagal baroreflex function, it is important to maintain lower central arterial stiffness.

Recently, statements on physical activity have recommended resistance training (RT) for muscular hypertrophy and maximizing strength [10, 11]. Several studies have reported that RT increases and decreases central arterial stiffness and compliance in healthy individuals, respectively [12–15]. On the other hand, García-Mateo et al. [16] have reported that RT, of at least four weeks duration and two days per week frequency, does not alter central arterial stiffness and compliance in systematic review. However, many studies have demonstrated that resistance-trained men who have performed for long duration training (> 2 years) at high-frequency (> 5 days/week), such as bodybuilders, throwers and weight-lifters, almost certainly have higher central arterial stiffness and lower arterial compliance than age-matched untrained men [17–21]. The central arterial stiffening in resistance-trained men may be associated with lower cardiovagal BRS.

The purpose of this study was to examine the effect of greater central arterial stiffness in resistance-trained men on cardiovagal BRS. Because resistance-trained men almost certainly have higher central arterial stiffness and lower arterial compliance than age-matched untrained men [17–21], a cross-sectional study comparing between the trained and untrained was appropriate to investigate the purpose of this study. Therefore, we designed a cross-sectional study in which central arterial stiffness, compliance, and cardiovagal BRS were compared between resistance-trained and age-matched untrained men. We hypothesized that greater central arterial stiffness in resistance-trained men would be associated with lower cardiovagal BRS. Although this phenomenon has not been considered a problem because the effect of central artery adaptation with RT on cardiovascular diseases has not been clarified yet, this study may provide evidence about the potential risk of central arterial stiffening with RT.

## Methods

### Participants

Forty healthy men (resistance-trained group; *n* = 20 and control group; *n* = 20) were recruited to participate in this study. The participants in the resistance-trained group had been performing RT for > 2 years, > 5 days/week. Moreover, participants in the resistance-trained group performed RT and used free weights and machines. Furthermore, these participants were in a training period for muscle hypertrophy in this study. None of the participants in the control group regularly engaged in RT and performed other exercise training < 3 days/week. All of the participants were normotensive (< 130/80 mmHg), had no history of cardiovascular diseases, diabetes, smoking, and were not taking medications such as anabolic steroids.

All of the participants provided written informed consent to participate prior to the start of the study. All of the procedures and risks of this study were reviewed and approved by the Human Research Committee of Waseda University (approval No. 2019-102). The study adhered to the principles of the Declaration of Helsinki.

### Protocol

The studies were performed following a 3-h fast. The participants were required to avoid and caffeine intake for at least 12 h and alcohol intake for at least 24 h before participating in the study. The participants were also evaluated 24 h after their last exercise session to avoid the acute effects of exercise.

All participants underwent measurement of hemodynamic and carotid arterial variables, and cardiovagal BRS in a temperature- and humidity-controlled environment (Temperature: 22.0 ± 0.1 °C, Humidity: 50.0 ± 0.3%) after 15 min in a resting supine position. All measurements were carried out under comfortable laboratory conditions between 09:00 AM and 1:00 PM.

### Body Composition

Body composition was measured using bioelectrical impedance analysis (InBody 720, InBody Japan Inc., Tokyo, Japan) with the participant in the upright position.

### Muscular Strength

Handgrip strength was measured using a grip dynamometer (Grip-D, Takei Scientific Instruments Co., Ltd., Tokyo, Japan) as an index of muscular strength with the participant in the standing position. The participants were instructed to stand and extend their dominant hand by their sides during a hand grip execution and to grip the dynamometer with full effort for three seconds. The values (kg) were calculated as the average of two trials.

### Central Arterial Stiffness and Compliance

The β-stiffness index and arterial compliance of the carotid artery were measured as an index of central arterial stiffness and compliance, respectively. Both the β-stiffness index and arterial compliance were measured in the right carotid artery using a combination of a brightness mode ultrasonography system for the carotid artery diameter and applanation tonometry for the carotid blood pressure (BP). The carotid artery diameter was obtained 1.0–2.0 cm proximal to the carotid bifurcation using an ultrasonography system equipped with a 10-MHz linear transducer (LOGIQ-e, GE Medical Systems, Tokyo, Japan). The diameter was recorded over ten cardiac cycles with the brightness mode in the longitudinal section. The images obtained were analyzed using image analysis software (ImageJ, NIH, USA), and these images were used to analyze the systolic diameter (sD) and diastolic diameter (dD).

The carotid pressure waveform was obtained in the right carotid artery. The obtained pressure waveforms were converted from a pencil-type probe incorporating a high-fidelity strain-gauge transducer (SPT-301, Millar Instruments, TX, USA) at a sampling rate of 1000 Hz through an analog/digital converter (PowerLab/16SP, AD Instruments, New South Wales, Australia) and recorded in a device connected to a personal computer (MacBook, Apple, CA, USA). Then, the obtained data were analyzed using an analysis software (LabChart5, AD Instruments, New South Wales, Australia). The carotid arterial pressure was calibrated by equating the carotid diastolic blood pressure (DBP) and mean arterial pressure (MAP) to the brachial artery value [22]. The β-stiffness index and arterial compliance were calculated as follows [12, 13, 18, 19]:

$$\upbeta -\mathrm{stiffness index}= \frac{\mathrm{ln}(\mathrm{carotid systolic blood pressure }[\mathrm{SBP}]/\mathrm{DBP})}{(\mathrm{sD}-\mathrm{dD})/\mathrm{dD}}$$

$$\mathrm{Arterial compliance}=\frac{(\mathrm{sD}-\mathrm{dD})/\mathrm{dD}}{2(\mathrm{carotid SBP}-\mathrm{DBP})}\uppi {\mathrm{dD}}^{2}$$

### Carotid Arterial Intima-Media Thickness

The carotid arterial intima-media thickness (CA IMT) was measured 1.0–2.0 cm proximal to the carotid bifurcation with an ultrasonography system equipped with a 10-MHz linear transducer (LOGIQ-e, GE Medical Systems, Tokyo Japan). The obtained images were analyzed using image analysis software (ImageJ, NIH, USA). At least ten CA IMT measurements were taken, and the mean value was used for analysis [12, 13, 18, 19].

### Hemodynamics

HR and beat-to-beat ABP were acquired using a three-lead ECG (BSM-2401, NIHON KOHDEN, Japan) and finger photoplethysmography (Finapres Medical Systems, Amsterdam, The Netherlands), respectively. The photoplethysmograph was attached to the middle finger of the right hand. Additionally, stroke volume (SV) was calculated from the obtained ABP waveform using the Modelflow method [23, 24], which incorporates age, height and weight, and simulates aortic flow waveforms from an arterial pressure signal using a nonlinear three-element model of the aortic input impedance (Beatscope, version 1.1, Finapres Medical Systems, Amsterdam, The Netherlands). Cardiac output (CO) and total peripheral resistance (TPR) were then calculated as SV × HR and MAP / CO, respectively.

### Cardiovagal Baroreflex Sensitivity

**Fig. 1:**
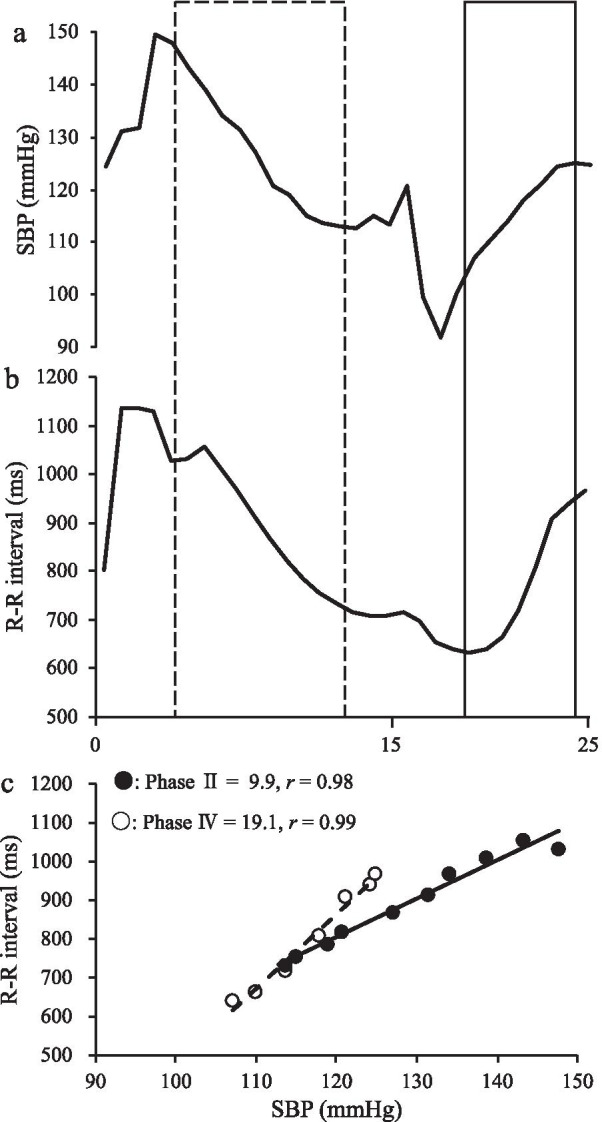
Typical responses of systolic blood pressure (SBP) (**a**) and R-R interval (**b**) during the Valsalva maneuver (VM). (**c**) shows a typical linear regression result between SBP and R-R interval during Phase II and IV of VM. Cardiovagal baroreflex sensitivity Phase II and Phase IV were estimated from outlined with a dashed and a solid line, respectively. SBP, systolic blood pressure; VM, Valsalva maneuver

The Valsalva maneuver (VM) is separated by four phases as described by Hamilton et al. [25]: (Phase I) onset of strain with an increase in ABP and a decrease in HR, (Phase II) continued strain with a decrease in ABP and its later partial recovery, (Phase III) strain release with a sudden decrease in ABP and further heart acceleration, and (Phase IV) system recovery with ABP overshoot. Cardiovagal BRS was evaluated using the slope of the R-R interval and systolic BP (SBP) during Phase II (Cardiovagal Phase II) and IV (Cardiovagal Phase IV) of the VM [2–4, 26, 27]. Figure 1 shows the typical responses of SBP (a) and R-R interval (b) during the VM.

VM was carried out to maintain an expiratory mouth pressure of 40 mmHg for 15 s by blowing through a short tube connected pressure gauge after deep inspiration in supine position. The pressure values were displayed to provide visual feedback to the participants. Immediately after, participants were instructed to maintain normal respiration and to avoid deep respiration. The waveform of the beat-to-beat R–R interval and SBP was digitally converted at a sampling rate of 1000 Hz through an analog/digital converter, recorded in a device connected to a personal computer, and analyzed with analysis software (LabChart 5, AD Instruments, New South Wales, Australia). Cardiovagal Phase II was estimated from what constitutes the first decrease in BP, from strain onset to strain termination. On the other hand, Cardiovagal Phase IV was evaluated from where the R–R interval began to lengthen and continued to the point of maximal SBP elevation. Then, the slope of linear correlation between the R–R interval and the SBP was assessed for both BRSs, which was determined if the *r* value was > 0.8, as previously described (Fig. 1c) [28]. Both BRSs were calculated using spreadsheet software (Microsoft Excel, Microsoft, WA, USA) immediately after each VM trials. The VM was performed until three acceptable values (*r* > 0.8) were obtained. As a result, all participants performed 5–10 VM trials at 3- to 5-min intervals. The average of the three values was taken as the definitive measurement.

### Data and Statistical Analysis

The cardiovagal BRS was not completely blindly analyzed because the index was calculated immediately after each VM trial to check whether acceptable values (*r* > 0.8) were obtained. However, all ultrasonography system data were randomly selected by a third person who was not involved in this study, and the selected data were then analyzed by authors who were unaware of which study participant the data belonged to.

All values were presented as mean ± standard deviation (SD). Statistical analyses were performed using statistical analysis software (SPSS version 26.0 for Mac, IBM, Tokyo, Japan). The Shapiro–Wilk test was performed to determine the normality of data parameters. The mean differences in the two groups were examined using the Student’s unpaired *t* test. To investigate the effects of central arterial stiffening in resistance-trained men on cardiovagal BRS, Pearson correlations were used to assess the relationship between cardiovagal BRS and β-stiffness index, and between cardiovagal BRS and arterial compliance. In all of the analyses, the level of significance for all comparisons was set at *P* < 0.05.

## Results

### Characteristics of the Participants

Table 1 shows the characteristics of the participants. Body weight, body mass index, lean body mass, and hand grip strength were significantly higher in the resistance-trained group than in the control group (*P* < 0.05). However, no significant differences were observed in the other characteristics of the participants between the two groups. The training frequency of the resistance-trained group and the control group was 5 ± 1 and 2 ± 1 days/week, respectively. Moreover, the overall training duration in the resistance-trained group was 3.8 ± 1.6 years.

**Table 1:** Characteristics of the participants in control and resistance-trained groups

	Control (*n* = 20)	Resistance-trained (*n* = 20)
Age (y)	25 ± 2	22 ± 3
Height (cm)	172 ± 6	173 ± 7
Body weight (kg)	70 ± 9	80 ± 9*
Body fat (%)	19 ± 5	17 ± 4
Body mass index (kg/m^2^)	23.7 ± 2.4	26.7 ± 2.2*
Lean body mass (kg)	56 ± 5	66 ± 7*
Handgrip (kg)	41 ± 5	48 ± 6*
Training duration (y)	–	3.8 ± 1.6
Training frequency (days/week)	1 ± 1	5 ± 1*

Values are mean ± SD

^*^P < 0.05 versus control group

### Hemodynamics and Carotid Arterial Variables

Table 2 shows hemodynamics and carotid arterial variables. The brachial DBP was significantly lower in the resistance-trained group (*P* < 0.05). Brachial and carotid pulse pressure (PP) were significantly higher in the resistance-trained group than in the control group (both, *P* < 0.05). However, no significant differences were observed in other hemodynamic and carotid arterial variables between the two groups at rest.

**Table 2:** Hemodynamics and carotid arterial variables in control and resistance-trained groups

	Control (*n* = 20)	Resistance-trained (*n* = 20)
Brachial SBP (mmHg)	110 ± 8	110 ± 6
Brachial DBP (mmHg)	61 ± 6	57 ± 4*
Brachial MAP (mmHg)	79 ± 6	78 ± 5
Brachial PP (mmHg)	49 ± 5	53 ± 4*
Carotid SBP (mmHg)	101 ± 8	104 ± 7
Carotid PP (mmHg)	40 ± 6	47 ± 7*
CA diameter (mm)	6.0 ± 0.5	6.1 ± 0.5
CA IMT (mm)	0.43 ± 0.03	0.43 ± 0.05
HR (bpm)	56 ± 7	58 ± 8
SV (ml)	96 ± 13	94 ± 16
CO (l/min)	5.4 ± 0.9	5.5 ± 1.2
TPR (mmHg/l/min)	15.1 ± 2.8	14.9 ± 3.6
Cardiovagal BRS Phase II (ms/mmHg)	7.6 ± 3.1	5.3 ± 2.4*

Values are mean ± SD

SBP, systolic blood pressure; DBP, diastolic blood pressure; MAP, mean arterial pressure; PP, pulse pressure; CA, carotid artery; IMT, intima-media thickness; HR, heart rate; SV, stroke volume; CO, cardiac output; TPR, total peripheral resistance; BRS, baroreflex sensitivity; VM, Valsalva maneuver

^*^P < 0.05 versus control group

### Central Arterial Stiffness and Compliance, Cardiovagal Baroreflex Sensitivity

**Fig. 2:**
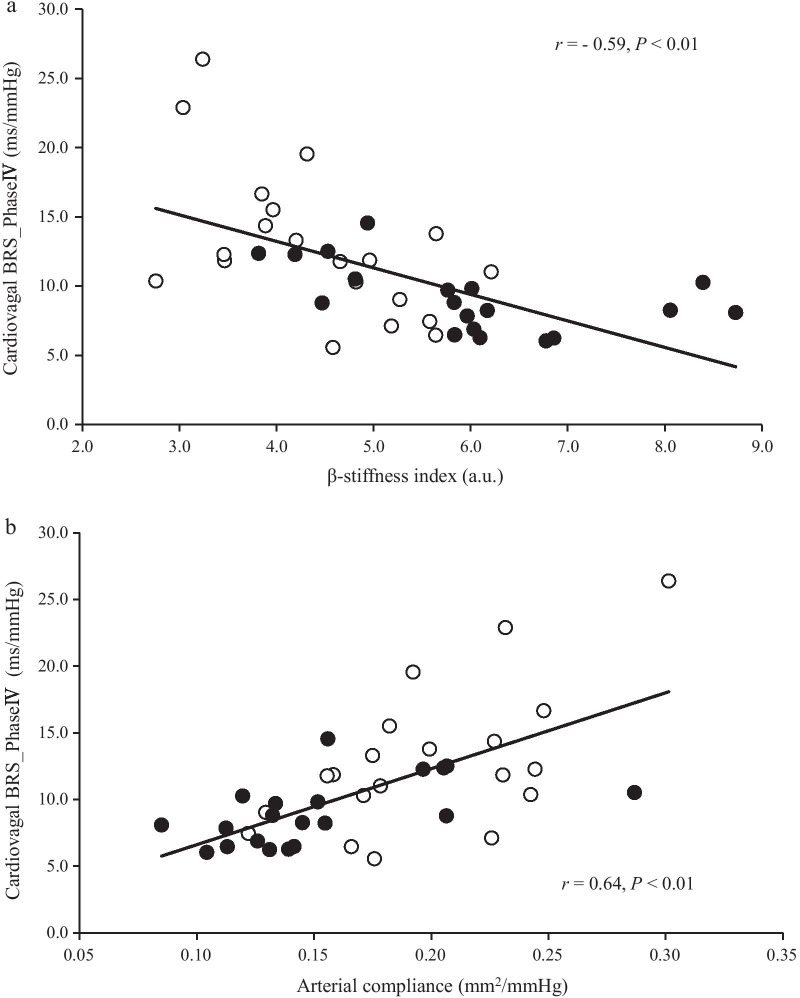
Linear regression between cardiovagal baroreflex sensitivity during the Phase IV of the Valsalva maneuver and β-stiffness index (**a**), arterial compliance (**b**). Open circle (○): control individuals, close circle (●): resistance-trained individuals

The β-Stiffness index was significantly higher in the resistance-trained group than in the control group (*P* < 0.01, Fig. 2a). By contrast, the resistance-trained group demonstrated significantly lower arterial compliance and cardiovagal BRS than the control group (*P* < 0.01, Fig. 2b and c, respectively).

### Relationship Between Cardiovagal Baroreflex Sensitivity and Central Arterial Mechanical Properties

**Fig. 3:**
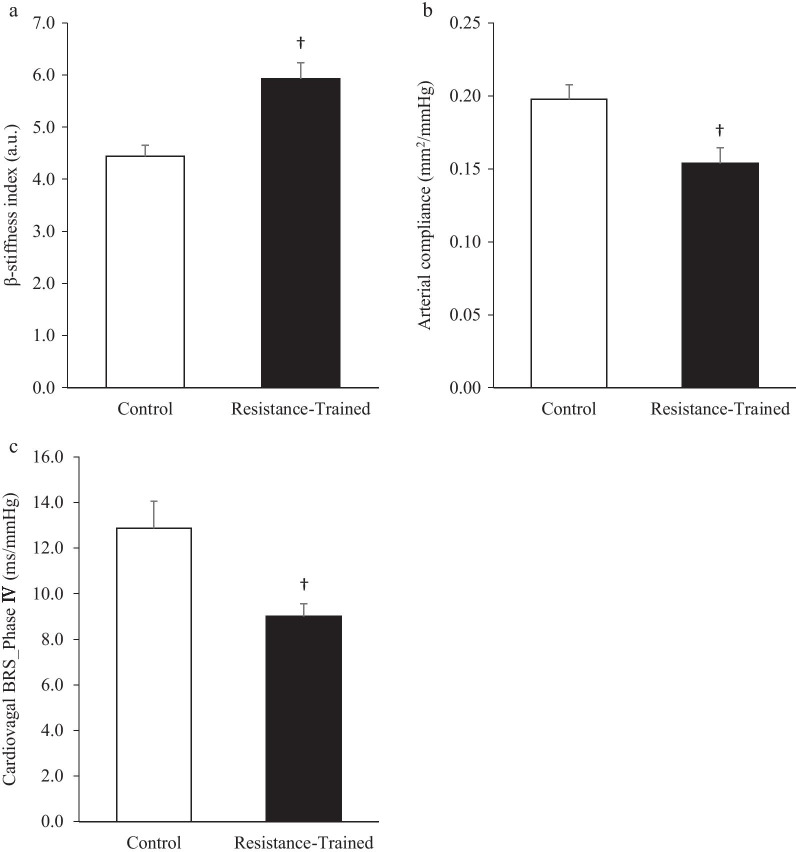
β-stiffness index (**a**) and arterial compliance (**b**) cardiovagal baroreflex sensitivity during the Phase IV of the Valsalva maneuver (c) in both groups. Values are mean ± SD. ^†^*P* < 0.01 versus control group

The associations between cardiovagal BRS and β-stiffness index, and between cardiovagal BRS and arterial compliance are shown in Fig. 3a and b, respectively. A negative correlation was found between cardiovagal BRS and the β-stiffness index (*r* = -0.59, *P* < 0.01), and a positive correlation was found between cardiovagal BRS and arterial compliance (*r* = 0.64, *P* < 0.01).

## Discussion

The present study showed that the resistance-trained group demonstrated a higher β-stiffness index (Fig. 3a), lower arterial compliance (Fig. 3b), and lower cardiovagal BRS (Fig. 3c) compared with the control group. In addition, cardiovagal BRS Phase IV was inversely and positively correlated with the β-stiffness index and arterial compliance, respectively (Fig. 2a, b). These findings suggest that greater central arterial stiffness in resistance-trained men may be associated with lower cardiovagal BRS.

### Characteristics of the Participants and Hemodynamics

Body weight, body mass index, lean body mass, and hand grip strength were significantly higher in the resistance-trained group than in the control group (Table 1). The results also considered the findings of previous studies that compared resistance-trained men with untrained men. However, the handgrip scores in the RT group were certainly not high. Most of the participants in the RT group had participated in a competition (i.e., bodybuilding competition, weight- or power-lifting) and achieved excellent grades. Therefore, the participants in the resistance-trained group were thought to be well-trained. Moreover, given that the RT group had a higher body weight, we did not examine RT-induced systematic hemodynamic differences (e.g., larger SV/CO). We speculate that the average high value was caused by outliers, i.e., three participants in the control group were retired endurance athletes from over 4 years ago. Although these participants did not habitually perform exercise at the time of the study, the values of SV and CO were still high. The CO value was significantly different when these participants were excluded from the analysis.

### Central Arterial Stiffening with Resistance Training

Several studies have shown that resistance-trained men have higher central arterial stiffness and lower compliance than age-matched untrained men [17–21]. Several factors increase central arterial stiffness and decrease compliance such as impaired vascular endothelial function and increased sympathetic vasoconstrictor tone [29–34].

Otsuki et al. [21] have suggested that higher central arterial stiffness and lower arterial compliance in RT athletes are associated with a higher plasma endothelin-1 (ET-1) concentration than in the age-matched untrained individuals and endurance athletes. ET-1 is produced by vascular endothelial cells and is a strong vasoconstrictor [35]. A study has reported that pulse wave velocity, as an index of central arterial stiffness, is increased by the intraarterial infusion of ET-1 [36]. These findings suggest that ET-1 may contribute to the central arterial stiffening in resistance-trained men.

Elevation of muscle sympathetic nerve activity (MSNA) causes vasoconstriction [37], resulting in increasing arterial stiffness [32]. MSNA and central arterial stiffness are higher in resistance-trained individuals than in endurance-trained individuals, and MSNA is positively correlated with central arterial stiffness [32]. Moreover, norepinephrine (NE) concentration in the blood and central arterial stiffness are increased with upper limb RT, but not with lower limb RT according to Okamoto et al. [14]. The study has also shown a positive correlation between the changes in NE concentration in the blood and central arterial stiffness. These results indicate that changes in sympathetic nerve activity are associated with central arterial stiffness following RT [14, 32].

Pressor response during acute resistance exercise may also be associated with the elevation of central arterial stiffness and reduction of arterial compliance. RT may alter the arterial load-bearing properties and thereby cause arterial stiffening, because acute resistance exercise dramatically elevates BP as high as 320/250 mmHg in resistance-trained individuals [38]. Ozaki et al. [15] have demonstrated that carotid arterial compliance is decreased in the high-intensity RT group, and the changes are correlated with SBP elevations during acute exercise sessions.

However, it is unclear whether the effects of these factors on higher central arterial stiffness and lower arterial compliance (Fig. 3a, b) were found in the present study. Also, evidence is limited regarding the mechanisms underlying the central arterial stiffening in resistance-trained men. Thus, further studies are needed to investigate the precise mechanism of increased central arterial stiffness with high-frequency RT for long duration.

A meta-analysis of randomized controlled trials focused low- to high-intensity has demonstrated that RT improves vascular endothelial function [39], which seems to be at odds with increases in arterial stiffness and/or reduced arterial compliance. On the other hand, a meta-analysis focused only high-intensity RT demonstrated that the RT is associated with an increase in arterial stiffness in young participants [40]. Considering this context, training intensity is important factor whether increases in central arterial stiffness with RT. In addition, García-Mateo et al. [16] suggested that RT frequency and duration were associated with increases in central arterial stiffness in a systematic review. Otsuki et al. [21] demonstrated that resistance-trained men who perform high-intensity RT at high-frequency (5.1 ± 0.1 days/week) for long duration (> 2 years) have greater central arterial stiffness and plasma ET-1 levels, although the plasma nitrite/nitrate levels remain the same. A previous study showed that intraarterial infusion of exogenous ET-1 in young, apparently healthy individuals caused a significant attenuation of vascular endothelial function [41]. Thus, resistance-trained men may have impaired vascular endothelial function due to an elevation plasma ET-1 levels. On the other hand, it remains unclear whether the frequency and duration of high-intensity RT influence vascular endothelial function.

Although the resistance-trained group had a higher central arterial stiffness and lower central arterial compliance compared with the control group, no significant difference was observed in TPR (Table 2). Beck et al. [42] have demonstrated that high-intensity RT (approximately 70–80% 1RM) for 8 weeks reduces peripheral arterial stiffness. Considering the previous finding, we speculate that the reduction of peripheral arterial stiffness with high-intensity RT is a compensatory adaptation to prevent the elevation of TPR. However, it is unclear whether resistance-trained men who participated in the present study have lower peripheral arterial stiffness because we did not measure peripheral arterial stiffness.

### Relationship Between Cardiovagal Baroreflex Sensitivity and Central Arterial Mechanical Properties

The present study found a negative correlation between cardiovagal BRS and β-stiffness index, and a positive correlation between cardiovagal BRS and arterial compliance (Fig. 2a, b). These findings may support previous studies, which report that cardiovagal BRS is correlated with alteration of central arterial stiffness and compliance [2, 3, 7]. A previous animal study has found that low-intensity resistance training improves cardiovagal BRS [43]. Okamoto et al. [44] have reported that low-intensity resistance training decreases arterial stiffness. In other words, the different result with previous study may be influenced by training intensity. Intervention studies have also reported that high-intensity RT either decreases or did not change cardiovagal BRS in humans [45, 46]. These results may contradict with the partly same or completely different results of the present study. However, it is difficult to compare these results with our result because of the differences in the methods used to evaluate cardiovagal BRS. Although the present study used VM to evaluate cardiovagal BRS, previous studies utilized a more spontaneous methodology. Yang et al. [47] have demonstrated that spontaneous cardiovagal BRS was not correlated to the cardiovagal BRS of the VM. The authors also discussed that assessing cardiovagal BRS under different physiological conditions might be influenced by the different aspects of cardiovagal baroreflex function, which are likely due to the static (spontaneous) versus rapidly changing (VM) conditions. We noted that the spontaneous cardiovagal BRS was not significantly different between the groups. This result may support the finding of a previous study, suggesting that cardiovagal BRS is different when assessed with the VM [47]. However, the spontaneous cardiovagal BRS, which was estimated for 3 min at rest, was inadequate in the present study because previous studies have described that the index is obtained with the R–R interval and SBP for more than 10 min at rest [47]. Therefore, further studies are required to clarify the effect of central arterial stiffening with RT on cardiovagal BRS in both methodologies.

Arterial baroreceptors such as the aortic arch and carotid sinus constantly monitor ABP and the information is fed back to the medulla oblongata through the baroreceptive afferents nerve [47]. The ABP is then modified by an alteration of CO and peripheral vascular resistance via the autonomic nervous system [48]. The arterial baroreflex is associated with central arterial mechanical properties such as stiffness and compliance [2, 3, 7], because the arterial baroreceptors primarily sense the deformation of the arterial wall [49]. A previous study has also found that alteration of the arterial diameter is related to neural firing rather than ABP [8]. Based on these results, deformation of the arterial wall may be inhibited by stiffer artery in resistance-trained men compared with age-matched untrained men.

The cardiovagal BRS is determined not only by the central arterial mechanical properties but also by the neural component [50]. In an animal study, exercise training has been confirmed to attenuate sympathoexcitation through alterations in the GABAergic neurotransmission at the level of the nucleus tractus solitarius [50]. The neural transmission in the brain might have affected the cardiovagal BRS, which was assessed during Phase IV of VM similar to the present study. Even so, the present study did not assess the neural component. Therefore, further studies should be conducted to investigate the arterial baroreceptor-related neural component in resistance-trained men.

Cardiovagal BRS Phase II (Table 2) or IV (Fig. 3c) in the resistance-trained group was higher compared with those with diseases such as coronary artery disease [51], autonomic failure [52] and Parkinson’s disease [53]. However, the lower cardiovagal BRS in the resistance-trained group than in the control group should not be taken lightly. In contrast, considering the number of functional and physiological benefits with RT, RT should not be discouraged. Kawano et al. [12] demonstrated that combined moderate-intensity aerobic and high-intensity RT increases muscular strength and does not induce central arterial stiffening. Therefore, this strategy may prevent the reduction in cardiovagal BRS while keeping the benefits of RT.

### Study Limitation

There are several potential limitations in the present study. First, we recruited only men in both groups. A previous study has shown that high-intensity RT increases central arterial stiffness in women [54]. However, the female sex hormone estrogen also influences central arterial stiffness [55]. Therefore, because of the relative difficulty in separating female sex hormone contribution from the effect of RT on central arterial stiffening, women were not included in present study. Second, the participants in the present study were all young. Several studies have shown that central arterial mechanical properties and cardiovagal BRS are impaired by advancing age [2, 3, 7]. The interaction between age and RT on cardiovagal BRS should be examined in future studies. Finally, the present study was designed as a cross-sectional study that compared resistance-trained men and untrained men. Although habitual physical and untrained activity patterns also influence cardiovagal BRS [56, 57], these were not measured nor controlled in the present study. Therefore, this study’s findings should be confirmed with an intervention study.

## Conclusions

β-Stiffness index was significantly higher in the resistance-trained group than in the control group (Fig. 3a). By contrast, the arterial compliance and cardiovagal BRS in the resistance-trained group were significantly lower than those in the control group (Fig. 3b and c). Moreover, a negative correlation was found between cardiovagal BRS and β-stiffness index (Fig. 2a). Considering these results, greater central arterial stiffness in resistance-trained men may be associated with lower cardiovagal BRS. In light of the functional and physiological benefits with RT, the properly prescribed RT should still be highly recommended. The present study only raises caution when heavy and strenuous RT is to be prescribed.

## Data Availability

The data used to support the findings of this study are available from the corresponding author upon request.

## Abbreviations

ABP: Arterial blood pressure
BP: Blood pressure
BRS: Baroreflex sensitivity
CA: Carotid artery
CO: Cardiac output
DBP: Diastolic blood pressure
dD: Diastolic diameter
ECG: Electrocardiogram
ET-1: Endothelin-1
HR: Heart rate
IMT: Intima-media thickness
MAP: Mean arterial pressure
MSNA: Muscle sympathetic nerve activity
PP: Pulse pressure
RT: Resistance training
SBP: Systolic blood pressure
sD: Systolic diameter
SV: Stroke volume
TPR: Total peripheral resistance
VM: Valsalva maneuver

## References

[1] Laitinen T, Hartikainen J, Vanninen E, Niskanen L, Geelen G, Länsimies E (1985). Age and gender dependency of baroreflex sensitivity in healthy subjects. J Appl Physiol.

[2] Monahan KD, Dinenno FA, Seals DR, Clevenger CM, Desouza CA, Tanaka H (2001). Age-associated changes in cardiovagal baroreflex sensitivity are related to central arterial compliance. Am J Physiol Heart Circ Physiol.

[3] Monahan KD, Dinenno FA, Tanaka H, Clevenger CM, DeSouza CA, Seals DR (2000). Regular aerobic exercise modulates age-associated declines in cardiovagal baroreflex sensitivity in healthy men. J Physiol.

[4] Monahan KD, Tanaka H, Dinenno FA, Seals DR (2001). Central arterial compliance is associated with age- and habitual exercise related differences in cardiovagal baroreflex sensitivity. Circulation.

[5] Billman GE, Schwartz PJ, Stone HL (1982). Baroreceptor reflex control of heart rate: a predictor of sudden cardiac death. Circulation.

[6] Cerati D, Schwartz PJ (1991). Single cardiac vagal fiber activity, acute myocardial ischemia, and risk for sudden death. Circ Res.

[7] Mattace-Raso FU, van den Meiracker AH, Bos WJ, van der Cammen TJ, Westerhof BE, Elias-Smale S (2007). Arterial stiffness, cardiovagal baroreflex sensitivity and postural blood pressure changes in older adults: the Rotterdam Study. J Hypertens.

[8] Aars H (1969). Relationship between aortic diameter and aortic baroreceptor activity in normal and hypertensive rabbits. Acta Physiol Scand.

[9] Knutsen RH, Beeman SC, Broekelmann TJ, Liu D, Tsang KM, Kovacs A (2018). Minoxidil improves vascular compliance, restores cerebral blood flow, and alters extracellular matrix gene expression in a model of chronic vascular stiffness. Am J Physiol Heart Circ Physiol.

[10] American College of Sports Medicine (2009). American College of Sports Medicine Position stand Progression Models in Resistance Training for Healthy Adults. Med Sci Sports Exerc.

[11] Williams MA, Haskell WL, Ades PA, Amsterdam EA, Bittner V, Franklin BA, American Heart Association Council on Clinical Cardiology; American Heart Association Council on Nutrition, Physical Activity, and Metabolism (2007). Resistance exercise in individuals with and without cardiovascular disease: 2007 update: a scientific statement from the American Heart Association Council on Clinical Cardiology and Council on Nutrition, Physical Activity, and Metabolism. Circulation.

[12] Kawano H, Tanaka H, Miyachi M (2006). Resistance training and arterial compliance: keeping the benefits while minimizing the stiffening. J Hypertens.

[13] Miyachi M, Kawano H, Sugawara J, Takahashi K, Hayashi K, Yamazaki K (2004). Unfavorable effects of resistance training on central arterial compliance: a randomized intervention study. Circulation.

[14] Okamoto T, Masuhara M, Ikuta K (2009). Upper but not lower limb resistance training increases arterial stiffness in humans. Eur J Appl Physiol.

[15] Ozaki H, Yasuda T, Ogasawara R, Sakamaki-Sunaga M, Naito H, Abe T (2013). Effects of high-intensity and blood flow-restricted low-intensity resistance training on carotid arterial compliance: role of blood pressure during training sessions. Eur J Appl Physiol.

[16] García-Mateo P, García-de-Alcaraz A, Rodríguez-Peréz MA, Alcaraz-Ibáñez M (2020). Effects of resistance training on arterial stiffness in healthy people: a systematic review. J Sports Sci Med.

[17] Bertovic DA, Waddell TK, Gatzka CD, Cameron JD, Dart AM, Kingwell BA (1999). Muscular strength training is associated with low arterial compliance and high pulse pressure. Hypertension.

[18] Kawano H, Tanimoto M, Yamamoto K, Sanada K, Gando Y, Tabata I (2008). Resistance training in men is associated with increased arterial stiffness and blood pressure but does not adversely affect endothelial function as measured by arterial reactivity to the cold pressor test. Exp Physiol.

[19] Nakamura N, Muraoka I (2018). Resistance training augments cerebral blood flow pulsatility: cross-sectional study. Am J Hypertens.

[20] Otsuki T, Maeda S, Iemitsu M, Saito Y, Tanimura Y, Ajisaka R, Miyauchi T (2007). Relationship between arterial stiffness and athletic training programs in young adult men. Am J Hypertens.

[21] Otsuki T, Maeda S, Iemitsu M, Saito Y, Tanimura Y, Ajisaka R, Miyauchi T (2007). Vascular endothelium-derived factors and arterial stiffness in strength- and endurance-trained men. Am J Physiol Heart Circ Physiol.

[22] Armentano R, Megnien JL, Simon A, Bellenfant F, Barra J, Levenson J (1995). Effects of hypertension on viscoelasticity of carotid and femoral arteries in humans. Hypertension.

[23] van Lieshout JJ, Toska K, van Lieshout EJ, Eriksen M, Walløe L, Wesseling KH (2003). Beat-to-beat noninvasive stroke volume from arterial pressure and Doppler ultrasound. Eur J Appl Physiol.

[24] Wesseling KH, Jansen JR, Settels JJ, Schreuder JJ (1985). Computation of aortic flow from pressure in humans using a nonlinear, three-element model. J Appl Physiol.

[25] Hamilton WF, Woodbury RA, Harper HT (1936). Physiologic relationships between intrathoracic, intraspinal and arterial pressures. J Am Med Assoc.

[26] Komine H, Sugawara J, Hayashi K, Yoshizawa M, Yokoi T (1985). Regular endurance exercise in young men increases arterial baroreflex sensitivity through neural alteration of baroreflex arc. J Appl Physiol.

[27] Greenlund LM, Cunningham HA, Tikkanen AL, Bigalke JA, Smoot CA, Durocher JJ, Carter JR (2020). Morning sympathetic activity after evening binge alcohol consumption. Am J Physiol Heart Circ Physiol.

[28] Palmero HA, Caeiro TF, Iosa DJ, Bas J (1981). Baroreceptor reflex sensitivity index derived from Phase 4 of the Valsalva maneuver. Hypertension.

[29] Bruno RM, Prenno G, Daniele G, Pucci L, Lucchesi D, Stea F (2012). Type 2 diabetes mellitus worsens arterial stiffness in hypertensive patients through endothelial dysfunction. Diabetologia.

[30] Gaballa MA, Jacob CT, Raya TE, Liu J, Simon B, Goldman S (1998). Large artery remodelling during aging: biaxial passive and active stiffness. Hypertension.

[31] Lind L, Sarabi M, Millgard J, Kahan T, Edner M (1999). Endothelium-dependent vasodilation and structural and functional changes in the cardiovascular system are dependent on age in healthy subjects. Clin Physiol.

[32] Smith MM, Buffington CA, Hamlin RL, Devor ST (2015). Relationship between muscle sympathetic nerve activity and aortic wave reflection characteristics in aerobic- and resistance- trained subjects. Eur J Appl Physiol.

[33] Taddi S, Virdis A, Mattei P, Ghiadoni L, Gennari A, Fasolo CB (1995). Aging and endothelial function in normotensive subjects and patients with essential hypertension. Circulation.

[34] Haynes WG, Ferro CJ, O’Kane KP, Somerville D, Lomax CC, Webb DJ (1996). Systemic endothelin receptor blockade decreases peripheral vascular resistance and blood pressure in humans. Circulation.

[35] McEniery CM, Qasem A, Schmitt M, Avolio AP, Cockcroft JR, Wilkinson IB (2003). Endothelin-1 regulates arterial pulse wave velocity in vivo. J Am Coll Cardiol.

[36] Wilkinson IB, Qasem A, Mceniery CM, Webb DJ, Avolio AP, Cockcroft JR (2002). Nitric oxide regulates local arterial distensibility in vivo. Circulation.

[37] Rowell LB (1993). Human cardiovascular control.

[38] MacDougall J, InJones NLMN, McComas AJ (1986). Morphological changes in human skeletal muscle following strength training and immobilization. Human muscle power.

[39] Ashor AW, Lara J, Siervo M, Celis-Morales C, Oggioni C, Jakovljevic DG, Mathers JC (2015). Exercise modalities and endothelial function: a systematic review and dose-response meta-analysis of randomized controlled trials. Sports Med.

[40] Miyachi M (2013). Effects of resistance training on arterial stiffness: a meta-analysis. Br J Sports Med.

[41] Nishiyama SK, Zhao J, Eray DW, Richardson RS (1985). Vascular function and endothelin-1: tipping the balance between vasodilation and vasoconstriction. J Appl Physiol.

[42] Beck DT, Martin JS, Casey DP, Braith RW (2013). Exercise training reduces peripheral arterial stiffness and myocardial oxygen demand in young prehypertensive subjects. Am J Hypertens.

[43] Gomes MFP, Borges ME, Rossi VA, Moura EOC, Medeiros A (2017). The effect of physical resistance training on baroreflex sensitivity of hypertensive rats. Arq Bras Cardiol.

[44] Okamoto T, Masuhara M, Ikuta K (2011). Effect of low-intensity resistance training on arterial function. Eur J Appl Physiol.

[45] Cooke WH, Carter JR (2005). Strength training does not affect vagal-cardiac control or cardiovagal baroreflex sensitivity in young healthy subjects. Eur J Appl Physiol.

[46] Collier SR, Kanaley JA, Carhart R, Frechette V, Tobin MM, Bennett N (2009). Cardiac autonomic function and baroreflex changes following 4 weeks of resistance versus aerobic training in individuals with pre-hypertension. Acta Physiol.

[47] Yang H, Carter JR (2013). Baroreflex sensitivity analysis: spontaneous methodology vs. Valsalva’s maneuver Clin Auton Res.

[48] Shi X, Wray DW, Formes KJ, Wang HW, Hayes PM, O-Yurvati AH (2000). Orthostatic hypotension in aging humans. Am J Physiol Heart Circ Physiol.

[49] Brown AM (1980). Receptors under pressure. An update on baroreceptors. Hypertension.

[50] Mueller PJ, Hasser EM (2006). Putative role of the NTS in alterations in neural control of the circulation following exercise training in rats. Am J Physiol Regul Integr Comp Physiol.

[51] Airaksinen KE, Hartikainen JE, Niemela MJ, Huikuri HV, Mussalo HM, Tahvanainen KU (1993). Valsalva manoeuvre in the assessment of baroreflex sensitivity in patients with coronary artery disease. Eur Heart J.

[52] Wada N, Singer W, Gehrking TL, Sletten DM, Schmelzer JD, Low PA (2014). Comparison of baroreflex sensitivity with a fall and rise in blood pressure induced by the Valsalva manoeuvre. Clin Sci (Lond).

[53] Oka H, Mochio S, Onouchi K, Morita M, Yoshioka M, Inoue K (2017). Cardiovascular dysautonomia in de novo Parkinson’s disease. J Neurol Sci.

[54] Cortez-Cooper MY, DeVan AE, Anton MM, Farrar RP, Beckwith KA, Todd JS, Tanaka H (2005). Effects of high intensity resistance training on arterial stiffness and wave reflection in women. Am J Hypertens.

[55] Ahimastos AA, Formosa M, Dart AM, Kingwell BA (2003). Gender differences in large artery stiffness pre- and post puberty. J Clin Endocrinol Metab.

[56] Sala R, Malacarne M, Pagani M, Lusini D (2015). Evidence of increased cardiac parasympathetic drive in subjects meeting current physical activity recommendations. Clin Auton Res.

[57] O’Brien MW, Johns JA, Dorey TW, Frayne RJ, Fowles JR, Mekary S, Kimmerly DS (2020). Meeting international aerobic physical activity guidelines is associated with enhanced cardiovagal baroreflex sensitivity in healthy older adults. Clin Auton Res.

